# Optimizing age-based targeting in ice cleat distribution programs for preventing winter fall injuries: a cost-effectiveness modeling study

**DOI:** 10.1186/s40621-025-00637-2

**Published:** 2025-11-13

**Authors:** Carl Bonander, Johanna Gustavsson, Ulf Strömberg, Mikael Svensson

**Affiliations:** 1 School of Public Health and Community Medicine, Institute of Medicine, University of Gothenburg, Gothenburg, Sweden; 2 Center for Societal Risk Research, Karlstad University, Karlstad, Sweden

**Keywords:** Economic evaluation, Injury prevention, Targeted intervention, Fall injuries, Anti-slip devices, Studded footwear

## Abstract

**Background:**

Fall injuries on ice and snow are a major public health problem in cold climates, placing a substantial seasonal burden on healthcare systems and affected individuals. To prevent such injuries, many Swedish municipalities have implemented programs that distribute ice cleats, typically restricted to adults aged ≥ 65. Evidence suggests these programs increase cleat use, reduce injuries, and are cost-effective. However, it remains unclear whether restricting distribution to older adults is more effective than broader or universal strategies. This study is the first to formally evaluate this question.

**Methods:**

We developed a cohort simulation model combining elements of the local average treatment effects framework and the health belief model to evaluate the cost-effectiveness of alternative ice cleat distribution strategies across age thresholds. The model incorporates age-specific injury risks, compliance, costs, and quality-adjusted life year losses, using input from behavioral surveys, register data on ice-related fall injuries, and published literature. It is calibrated to outcomes from real-world distribution programs. Cost-effectiveness was assessed from a societal perspective, defining optimal thresholds as those maximizing net monetary benefit and acceptable thresholds as those with > 50% probability of being cost-effective compared to no distribution in probabilistic sensitivity analyses.

**Results:**

Our primary analysis identified ≥ 55 years as the optimal eligibility threshold (acceptable range: 42–72). Sensitivity analyses indicated that universal distribution may be acceptable if cleats are purchased restrictively and targeted to non-users, but it is unlikely to be optimal. When assuming short-lived behavior change (≤ 2 years) or valuing costs from a healthcare perspective only, no distribution was preferable.

**Conclusion:**

Age-targeted ice cleat distribution appears more cost-effective than universal provision and preferable to no distribution, but current programs limited to older adults may be suboptimal. Extending eligibility to middle-aged adults could further improve cost-effectiveness from a societal perspective.

## Introduction

Falls are a leading cause of injury-related morbidity and healthcare use [1]. While indoor hip fractures in older adults are well studied [2, 3], outdoor falls often affect otherwise healthy individuals across a wider age range [4, 5], yet preventive measures remain under-researched [5]. In cold climates, slips on snow and ice account for a substantial share of these injuries [6–9]. In Sweden, about half of all outdoor pedestrian fall injuries are ice-related [6], creating a significant seasonal burden on individuals and the healthcare system.

Ice cleats are attachable anti-slip devices that improve grip on icy surfaces and reduce the risk of outdoor winter falls [5, 10, 11]. To address the seasonal rise in such injuries, about 80 of Sweden’s 290 municipalities have implemented programs distributing free (or subsidized) cleats to older adults [12]. Emerging evidence suggests these programs are a cost-effective complement to routine snow and ice maintenance, achieving about 40% coverage of target groups [12], increasing uptake [13], and reducing injury rates [14]. Given their low cost, prior economic evaluations indicate that benefits likely outweigh costs [14–16].

Current programs usually limit distribution to adults aged ≥ 65 [12], likely reflecting perceived needs, political considerations, cost-effectiveness, and practical constraints [17, 18]. Yet it is unclear whether this age-based targeting is preferable to universal distribution or whether older adults are the best group to prioritize. Several factors suggest benefits from extending distribution to younger age groups. Ice-related fall injury rates rise from about age 50, stabilize between 65 and 70, and decline thereafter [19]. Moreover, effectiveness depends not only on age-specific risks but also on compliance—that is, how many non-users adopt cleats when offered. Observational data show that older adults already use cleats at relatively high rates, while middle-aged adults underuse them relative to their injury risk [19].

Given these patterns, it is important to ask whether current age-based targeting constitutes an optimal design for ice cleat distribution programs. To address this, we develop a decision-analytic model that combines data on ice cleat effectiveness with age-specific behavior and attitudes to estimate heterogeneity in compliance and policy effects. This framework allows us to evaluate the cost-effectiveness of targeted versus universal distribution and to identify optimal age threshold for targeted distribution.

## Methods

We conducted a health economic evaluation using a decision-analytic model to project the long-term costs and health effects of different intervention strategies from a societal perspective. Our reporting follows the Consolidated Health Economic Evaluation Reporting Standards 2022 [20].

### Intervention, comparator, and setting

We evaluated ice cleat distribution programs that provide one free pair of ice cleats to all citizens aged $\:{a}_{i}\ge\:d$ compared with no distribution. Here, $\:d$ represents an age eligibility threshold for distribution, which we vary across modeled scenarios. The model assumes a one-time distribution by local authorities (e.g., municipalities) and is informed by data from a Swedish setting. Sweden is divided into 21 regions and 290 municipalities (median population size in 2024: 16 181). Municipalities are self-governing, funded by local income taxes, and responsible for essential public services, including local transport safety interventions.

### Decision-analytic model

**Fig. 1:**
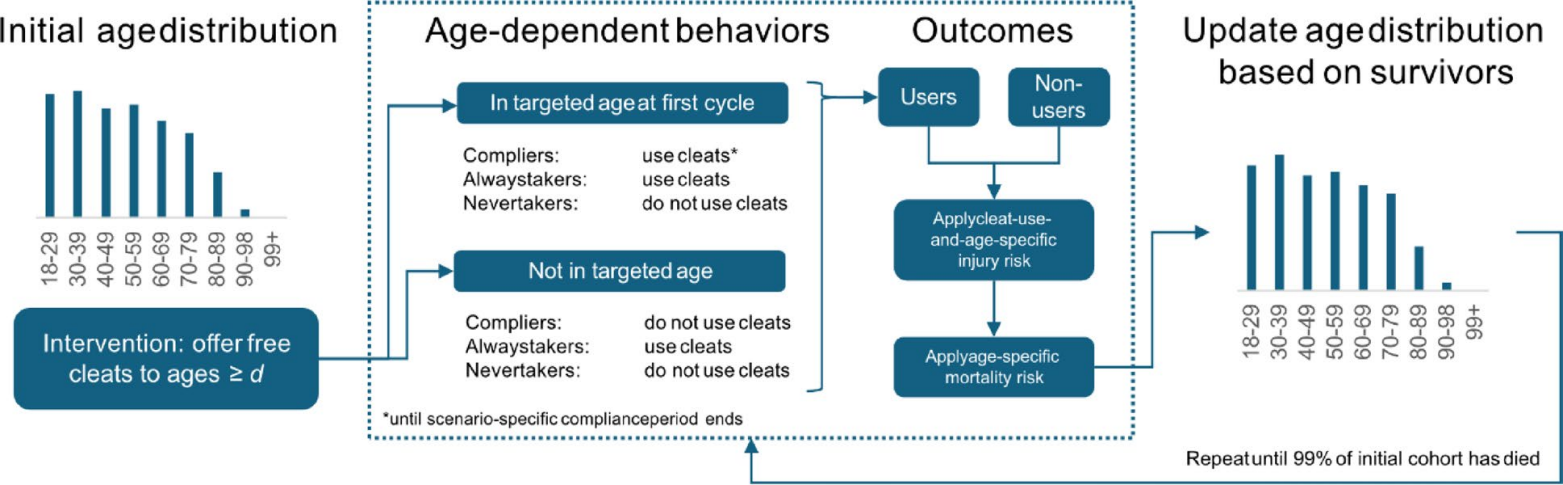
Simplified cohort flow in each annual cycle. At baseline the population is distributed across single-year ages. Individuals aged $\:\ge\:d$ (the distribution threshold) are offered free ice cleats. Compliers who are in these ages in the initial cycle adopt cleat use for a scenario-specific window (base-case: 3 years); always-takers already use cleats; never-takers never adopt. Age-specific injury and mortality risks are then applied to cleat users and non-users. Survivors age by one year, generating the age distribution for the next cycle

We developed a cohort simulation model to estimate the societal costs and effectiveness of these programs within a cohort of adults aged 18–99+ (Fig. 1). The cohort’s initial age profile reflects Sweden’s national age distribution in 2024 and is updated in one-year cycles until 99% of members have died, based on national mortality data from Statistics Sweden. Each year, survivors transition to the next age group, except those aged 99+, who remain in that group until death or model termination. The cohort is closed, meaning that no new individuals are added after the initial cycle. Because the distribution program is implemented only once at the start of the simulation, adding new individuals later would not change outcomes—those entering after the initial cycle would never be offered cleats and would therefore remain unexposed, just as in the no-distribution scenario.

Each year, cohort members face risks of ice-related fall injuries and all-cause mortality. Injury risks vary by age and cleat usage, while death risks vary by age. Due to limited data on long-term injury consequences following pedestrian falls, injuries are modeled with short-term health effects lasting one year; individuals recover fully in the next cycle unless reinjured. Death probabilities are not adjusted after ice-related fall injuries, as fatal outcomes are rare [15]. These assumptions are conservative, likely biasing estimates against ice cleat distribution.

Further technical details are provided in the Appendix. Model input parameters and sources are summarized in Table 1.

**Table 1:** Model input parameters, distributions, and data sources

Parameter	Mean	SE	Distribution (PSA)	Data source(s)
Annual injury rate	Age-specific	Age-specific	Poisson^a^	National Patient Register Total Population Register
Behavioral responses - Always-takers, $\:{p}_{i}^{A}$ - Never-takers, $\:{p}_{i}^{N}$ - Potential compliers, $\:{\stackrel{\sim}{p}}_{i}^{C}\:$	Age-specific	Age-specific	Multinomial^a^	National survey [15]
Compliance calibration, $\:\varphi\:$	0.708	0.127	Beta (8.31, 3.43)	[13]
Effect of ice cleat use, logRR	−0.799	0.333	Lognormal^a^	[10]
Program cost per ice cleat pair	€10.27	1.113	Gamma (85.17, 0.12)	[12,15]
Cost per injury	€6503	663.62^b^	Gamma (96.04, 67.72)	[21]
QALY decrement per injury	−0.1488	0.015^b^	Beta (81.60,466.79)	[14,21]

RR, Risk Ratio. PSA, Probabilistic Sensitivity Analysis. QALY, Quality−Adjusted Life Year. Costs are in 2025 Euros (€)

^a^Age−specific estimates are based on empirical data and posteriors from Bayesian models, see main text for details

^b^Empirical uncertainty estimates unavailable. Standard errors and distributional parameters set to approximately vary the parameter by ±20%

### Age-specific intervention effectiveness

The cohort is stratified into three behavioral groups following the local average treatment effects framework [21], which separates people based on how they respond to being offered ice cleats: (i) *always-takers* ($\:{p}_{i}^{A}$), who use cleats regardless of distribution; (ii) *never-takers* ($\:{p}_{i}^{N}$), who would never use them, and (iii) *compliers* ($\:{p}_{i}^{C}$), who use cleats only if offered a pair as part of a distribution program. These shares vary by age and updated annually as the cohort ages. Differences between the distribution and no-distribution scenarios arise solely from compliers, as all else is equal.

#### Compliance modelling

We estimate age-specific shares of *potential compliers*, which we denote by $\:{\stackrel{\sim}{p}}_{i}^{C}$, using data from a 2007 national survey by the Swedish Civil Contingencies Agency, which was sent to a random population sample of the adult Swedish population (18–79 years; *n* = 20 881 respondents, response rate: 62%). While later surveys by the same organization exist [13, 19], the 2007 survey is unique in that it included questions about self-reported use of anti-slip devices and attitudes toward their safety benefits.

Specifically, the survey asked, “*How often do you do the following things for your own safety?*” with one item being “*Use anti-slip devices in slippery road conditions (e.g.*,* ice cleats)*.” Response options ranged from *Never* to *Always* plus *Not applicable*. We coded those who responded *Sometimes*, *Most of the time*, and *Always* as ice cleat users and the rest as non-users. A second question asked, “*How important do you think the following measures are for increasing your safety?*” with the item “*Using anti-slip devices in slippery road conditions (e.g.*,* ice cleats)*.” Responses ranged from *Very unimportant* to *Very important* plus *Do not know*. We coded *Somewhat important* and *Very important* as indicating a positive safety belief.

Respondents who were non-users but expressed a positive safety belief were classified as potential compliers, capturing the perceived benefit construct of the Health Belief Model (HBM) [22]. Being offered a free pair can act as a cue to action and removes any potential cost barriers; however, because other HBM constructs (e.g., residual barriers, self-efficacy) are unobserved, we interpret $\:{\stackrel{\sim}{p}}_{i}^{C}$ as an upper bound for the behavior change following ice cleat distribution.

#### Calibration to empirical estimates

To align attitude-based compliance estimates with observed behavior change, we apply a calibration factor that scales the survey-based estimates to match the increase in cleat use observed among 65–79-year-olds in 63 Swedish municipalities following ice cleat distribution [13]. We define the compliance calibration factor as

$$\:\varphi\:=\frac{{\widehat{p}}_{obs}^{C}}{{\stackrel{\sim}{p}}_{65-79}^{C}},\:\:0\le\:\varphi\:\le\:1$$

where $\:{\widehat{p}}_{obs}^{C}$ denotes the observed average increase in cleat use among 65–79-year-olds reported by Holmberg et al. [13], and $\:{\stackrel{\sim}{p}}_{65-79}^{C}$ denotes the corresponding attitude-based estimate from the 2007 survey. We apply this factor uniformly across ages to obtain a calibrated compliance share that better reflects real-world, contemporary data on uptake:

$$\:{p}_{i}^{C}=\varphi\:\times\:{\stackrel{\sim}{p}}_{i}^{C},$$

The share of always-takers ($\:{p}_{i}^{A}$) is based on the age-specific prevalence of current cleat users from the same survey. Never-takers are the residual group: $\:{p}_{i}^{N}=1-{p}_{i}^{C}-{p}_{i}^{A}$.

#### Compliance dynamics over time

The program is implemented once at baseline, targeting all ages $\:{a}_{i}\ge\:d$. Only the compliers who are eligible at that time become realized users. In practice, cleat use may persist beyond the initial distribution through voluntary re-purchase or taper off over time due to non-adherence or device wear. While the long-term compliance dynamics are uncertain, our base-case model assumes that compliers continue to use cleats for three years—consistent with the average post-distribution follow-up duration in Holmberg et al. [13], from which the calibration factor $\:\varphi\:$ is derived. This factor thus indirectly incorporates real-world factors such as early abandonment and replacement. After three years, individuals either continue using cleats naturally as they age or revert to non-use, depending on age-specific probabilities.

#### Injury outcomes

We combine injury data (ICD-10 external cause code: W00 Falls due to ice and snow) from the National Patient Register (2001–2020) with age-specific population data from Statistics Sweden. The Swedish patient register includes inpatient and specialized outpatient care episodes with national coverage across Sweden [23, 24]. To avoid counting multiple visits, hospital transfers, and re-admissions attributable to the same fall injury more than once, we include only one care episode per individual per year.

The observed age-specific injury risk, $\:{r}_{i}^{obs}$, reflects the combined risk among users and non-users. Following the potential outcomes framework [25], this can be expressed as the weighted average of risks for ice cleat users and non-users:

$$\:{r}_{i}^{obs}={p}_{i}^{A}{r}_{i}^{1}+\left(1-{p}_{i}^{A}\right){r}_{i}^{0},$$

where $\:{r}_{i}^{1}$ and $\:{r}_{i}^{0}$ are the potential injury risks for users and non-users, respectively. To estimate the effect of cleat use, we rely on a randomized controlled trial from Wisconsin, USA that evaluated the impact of ice cleats on outdoor fall injuries among older adults (RR = 0.45, 95% CI: 0.23–0.85; *n* = 109, age range: 65–96 years) [10]. As age-specific estimates are unavailable, our base-case analysis assumes a constant relative risk across ages, which means that the potential injury risk for ice cleat users can be written as: $\:{r}_{i}^{1}=RR{r}_{i}^{0}$. As shown in the Appendix, we can then derive the injury risk for non-users, $\:{r}_{i}^{0}$, using the following formula:

$$\:{r}_{i}^{0}=\frac{{r}_{i}^{obs}}{RR{p}_{i}^{A}+1-{p}_{i}^{A}}$$

We use these expressions to estimate age- and time-specific injury risks under different distribution scenarios. Specifically, let $\:{r}_{i}\left(d,k\right)$ denote the expected injury risk at age $\:i$ in cycle $\:k$, given a program with distribution threshold $\:d$, which is given by:

$$\:{r}_{i}\left(d,k\right)=\left[{p}_{i}^{U}\left(d,k\right)\times\:RR+\left(1-{p}_{i}^{U}\left(d,k\right)\right)\right]{r}_{i}^{0},$$

where $\:{p}_{i}^{U}\left(d,k\right)$ is the share of ice cleat users at age *i* in simulation cycle *k* under the policy defined by distribution threshold $\:d$. This user share includes always-takers and any surviving compliers who were eligible and remain within their compliance window. The difference between$\:\:{r}_{i}\left(d,k\right)$ and the corresponding risk in the no-distribution scenario gives the injury reduction at that point in the cohort simulation. With this approach, effect heterogeneity arises solely from age differences in baseline risk and compliance (although we also perform sensitivity analyses allowing for age-varying relative risk reductions, as detailed below). Formal expressions for these calculations, including the user share dynamics over time, are provided in the Appendix.

### Cost-effectiveness Estimation

#### Quality-adjusted life years

We quantify injury consequences as the expected loss of quality-adjusted life years (QALYs), applying a one-year QALY decrement (loss) per injury. This approach follows Eklund et al. [14] and reflects the limited availability of long-term follow-up data on QALY losses following outdoor pedestrian falls. Health-related quality of life estimates are based on EQ-5D questionnaires from Olofsson et al. [26], who surveyed 256 individuals (mean age: 64 years) treated in emergency departments for pedestrian fall injuries. Respondents reported retrospectively on their pre-injury quality of life (0.93) and at one day (0.207), two weeks (0.607), two months (0.715), and six months (0.798) after the injury. These were integrated using the trapezoidal rule to estimate the area under the curve for a one-year period, assuming recovery to baseline (0.93) at twelve months.

#### Costs

All costs are reported in 2025 Euros (€). Historical costs were first updated using the Swedish Consumer Price Index (May, 2025 SEK) and converted from SEK to Euros using the June 24, 2025 exchange rate (0.09 €/SEK).

Intervention cost data were taken from Bonander et al. [15], who—based on survey data from 34 Swedish municipalities with ice cleat programs and complete purchase records [12]—estimated total program costs, including procurement, distribution, and administration, at €10.27 per purchased ice cleat pair. We used this unit cost to calculate program costs in each scenario by multiplying it by the number of individuals above the distribution threshold in the initial cycle, assuming one pair is purchased per eligible person.

Costs per pedestrian fall injury are based on Olofsson et al. [26]. The estimate reflects a broad set of societal cost consequences: direct healthcare costs for treatment and rehabilitation; any prescription drug costs (medication); physical therapy costs; formal home care by help services, home healthcare, or personal assistance; informal (unpaid) home care by relatives; productivity loss; and transportation costs.

#### Cost-effectiveness outcomes

The optimal distribution strategy was defined as the age threshold yielding the highest net monetary benefit (NMB) compared with no distribution, provided that at least one option had a positive NMB. For each threshold $\:d$, we compute:

$$\:NMB\left(d,\lambda\:\right)={\Delta\:}Q\left(d\right)\times\:\lambda\:-{\Delta\:}C\left(d\right),$$

where $\:{\Delta\:}Q\left(d\right)$ is the total QALY difference, $\:{\Delta\:}C\left(d\right)$ the total cost difference, and $\:\lambda\:$ the maximum willingness-to-pay per QALY. We set $\:\lambda\:$ to 500,000 SEK (€45,000), consistent with the cost-effectiveness threshold often applied in Sweden. Costs and QALYs were discounted at 3% annually to account for time preferences, following standard health technology assessment guidelines [27].

### Uncertainty quantification and sensitivity analyses

We performed 1,000 probabilistic sensitivity analyses iterations to quantify uncertainty. Parameter uncertainty for fixed (non–age-dependent) characteristics was based on published estimates and standard errors (Table 1); for parameters where empirical standard errors were unavailable, we varied the parameter ± 20% around the base-case estimate. Thresholds with > 50% probability of being cost-effective relative to no distribution were defined as *acceptable*.

#### Models for empirical data and associated uncertainty

Bayesian models were used to smooth age-specific data and quantify uncertainty. For behavioral data, we also used these models to impute expected behavior shares for ages 80–99, which were not included in the national survey. Models were estimated using Integrated Nested Laplace Approximation (INLA) in R-INLA [28, 29], with default priors. To propagate uncertainty, we repeatedly sampled from the posterior distributions.

**Fig. 2:**
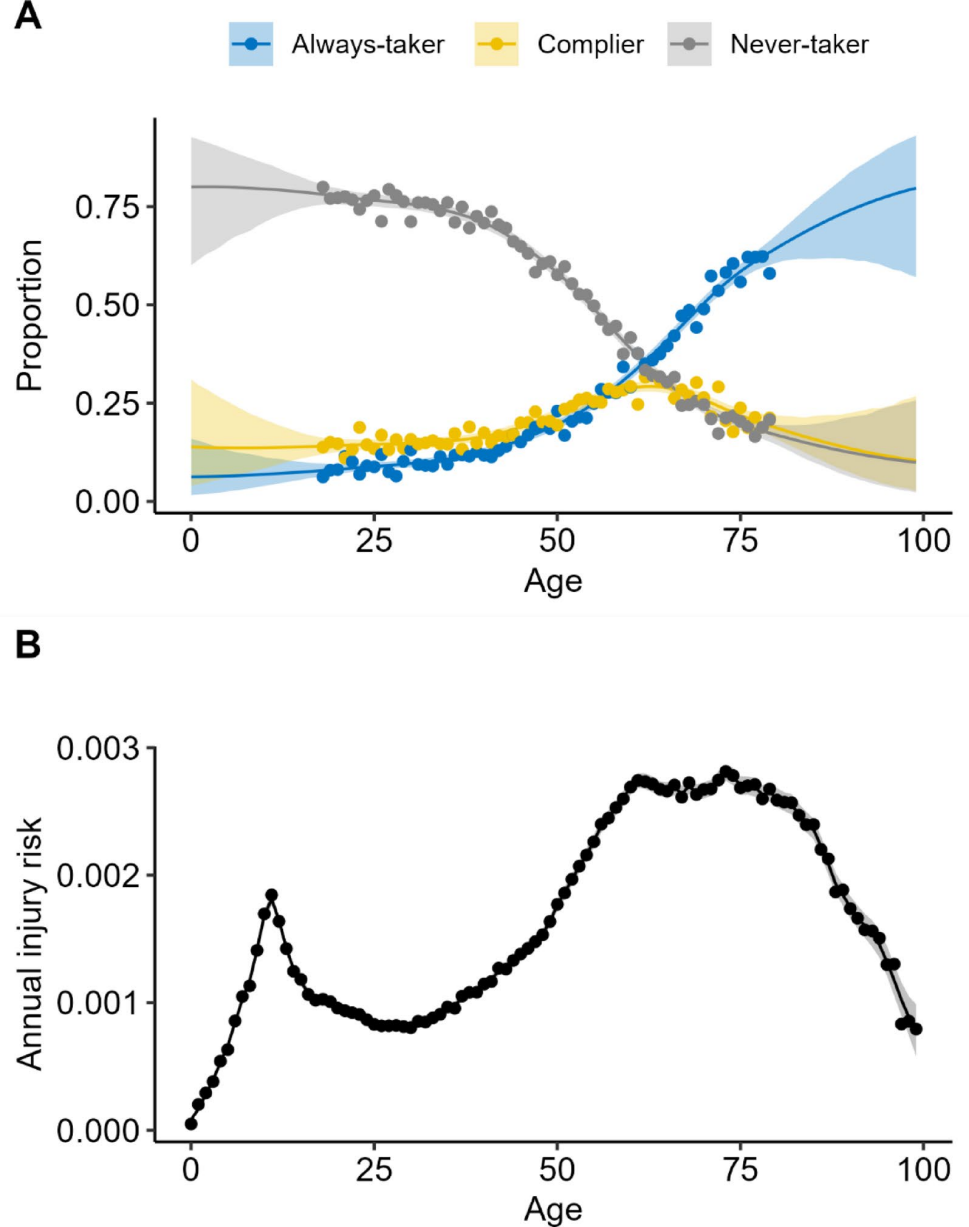
**A***:* Age-specific behavioral data on ice cleat use and potential compliance for ice cleat distribution (based on survey data for ages 18–79). Always-takers report already using ice cleats, never-takers report not using ice cleats and have a negative attitude toward their individual safety benefits, and compliers are non-users with a positive attitude toward their safety benefits. Shares reflect reported use and attitudes and are not calibrated in the plot. **B**: Age-specific annual injury risks for snow-and-ice-related falls based on specialized outpatient and inpatient data from the National Patient Register. Smooth lines and 95% credible intervals are based on Bayesian models with second-order random walks on age

For injury outcomes, we fitted a Bayesian Poisson model with a second-order random walk on age, which gives a smooth age curve. For behavioral data, we fitted a multinomial regression model using the Poisson trick [30]—a standard method allowing multinomial data to be analyzed with Poisson-based tools. Separate smooth age trends were estimated for always-takers, never-takers, and compliers, with predicted probabilities normalized to sum to one for each age. Model fits and observed data are shown in Fig. 2.

#### Deterministic sensitivity analyses

We conducted deterministic sensitivity analyses to test the robustness of our findings to structural assumptions. These included (i) an alternative, more optimistic program design in which cleats are procured for 50% of the eligible population, assuming that all compliers are still successfully reached while supplies last; (ii) alternative compliance durations of 2 and 4 years; (iii) discount rates of 1% and 5%; and (iv) a healthcare cost perspective excluding productivity losses and informal home care from injury costs.

In addition to these scenario analyses, we also examined how age heterogeneity might influence results by varying all age-invariant parameters using multiplicative factors by ± 1% and ± 2.5% per one-year age group. These factors were applied relative to the mean age in the original data source for parameters lacking age-specific data (QALY decrements, injury costs, log relative risk reduction from ice cleat use, and the compliance calibration factor). This analysis provides a test of how strongly the results would change if these parameters varied systematically with age.

### Model validation

Before conducting the analyses, we validated the model against external empirical estimates from Eklund et al. [14], a quasi-experimental study of 73 Swedish municipalities that distributed ice cleats to adults 65 + with 3.5 years of follow-up. Our model estimates a 12.0% reduction in ice-related fall injuries over three years for this age group, closely matching their observed estimate of 12.5% for programs distributing one pair per citizen. This close agreement suggests that the model is well-calibrated to real-world outcomes.

## Results

**Fig. 3:**
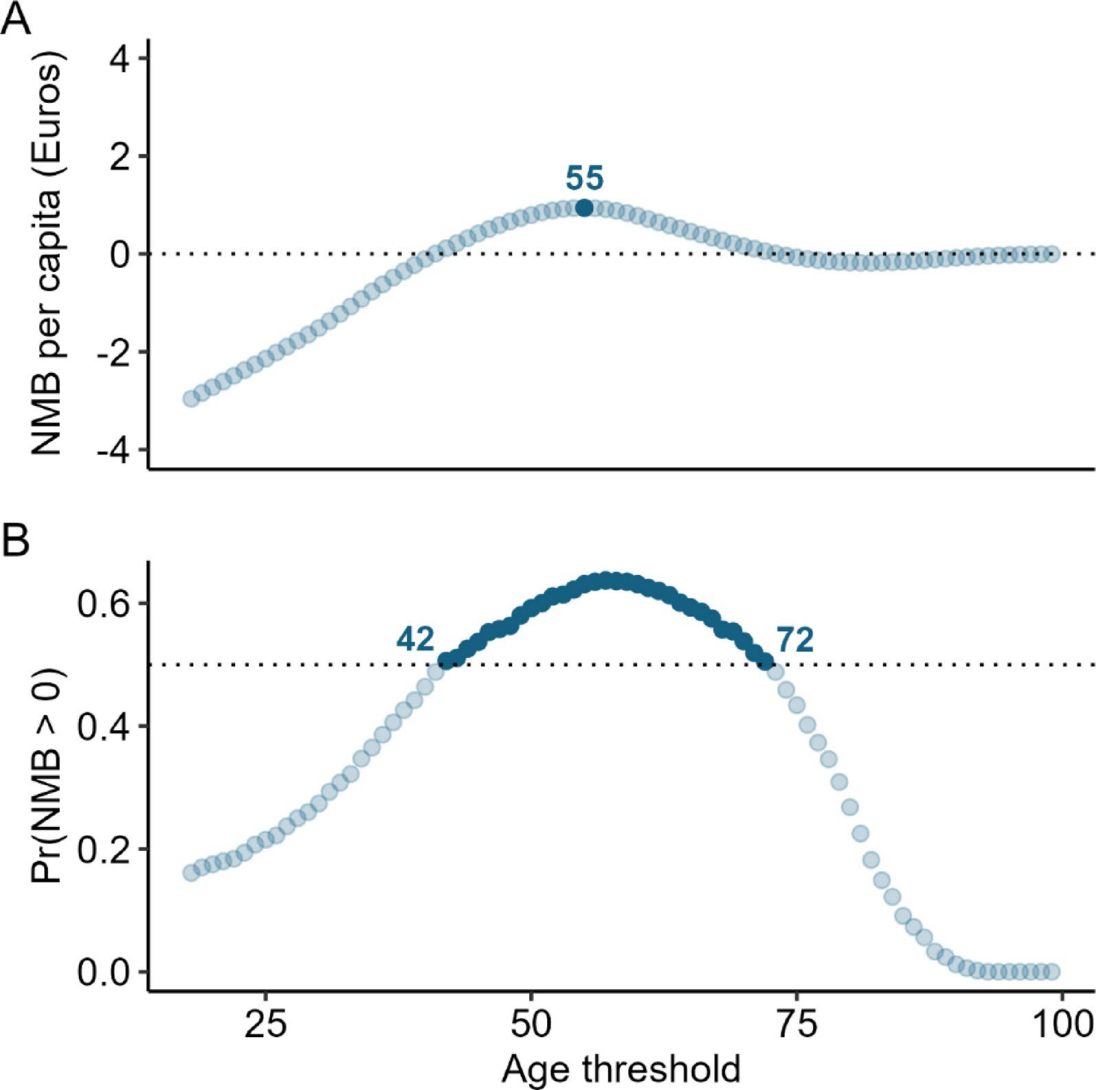
**A**: Estimated net monetary benefits of ice cleat distribution by age threshold (18–99+) compared to no distribution. The optimal threshold, which maximizes net benefit, is highlighted. **B**: Range of age thresholds where more than 50% of 1,000 iterations in the probabilistic sensitivity analysis indicate a positive net benefit. All highlighted choices in this range, while not necessarily optimal, are estimated to be preferable to no distribution

The base-case results suggest that targeted ice cleat distribution is cost-effective at several age thresholds compared with no distribution, with the optimal threshold estimated at ≥ 55 years (Fig. 3A). Probabilistic sensitivity analysis indicates that any threshold between ≥ 42 and ≥ 72 years has a greater than 50% chance of being cost-effective relative to no distribution (Fig. 3B), highlighting a range of acceptable—though not necessarily optimal—options. Overall, the results support targeted over universal distribution and suggest that extending eligibility to age groups below the commonly used ≥ 65 threshold may improve cost-effectiveness.

### Sensitivity analyses

Table 2 presents the estimated optimal age thresholds and acceptable threshold ranges under alternative scenarios. Assuming more efficient distribution (i.e., purchasing one pair for every two eligible individuals) lowers the optimal threshold to ≥ 47 years, with a broader acceptable range of 18–85 years. Extending the compliance duration to four years also lowers the optimal threshold to ≥ 51 years (range: 25–79 years), while shortening the compliance period to two years results in no distribution being preferred. Similarly, limiting the analysis to direct healthcare costs leads to no distribution being cost-effective. Varying the discount rate to 1% or 5% has no impact on the results.

**Table 2:** Optimal age thresholds for ice cleat distribution and acceptable ranges under different scenarios

Scenario	Optimal age threshold	Acceptable range
Base-case	≥ 55	42–72
More efficient distribution	≥ 47	18–85
Compliance − 1 year	N/A	N/A
Compliance + 1 year	≥ 51	26–78
Discount 5%	≥ 55	43–71
Discount 1%	≥ 55	41–73
Healthcare perspective	N/A	N/A

Acceptable range refer to the minimum and maximum age threshold where more than 50% of iterations in the probabilistic sensitivity analysis indicate a positive net monetary benefit compared to no distribution. N/A means that no distribution is preferred. More efficient distribution refers to a case where the programs purchase ice cleats more selectively (one ice cleat pair per every other eligible citizen), while still managing to reach all compliers

**Fig. 4:**
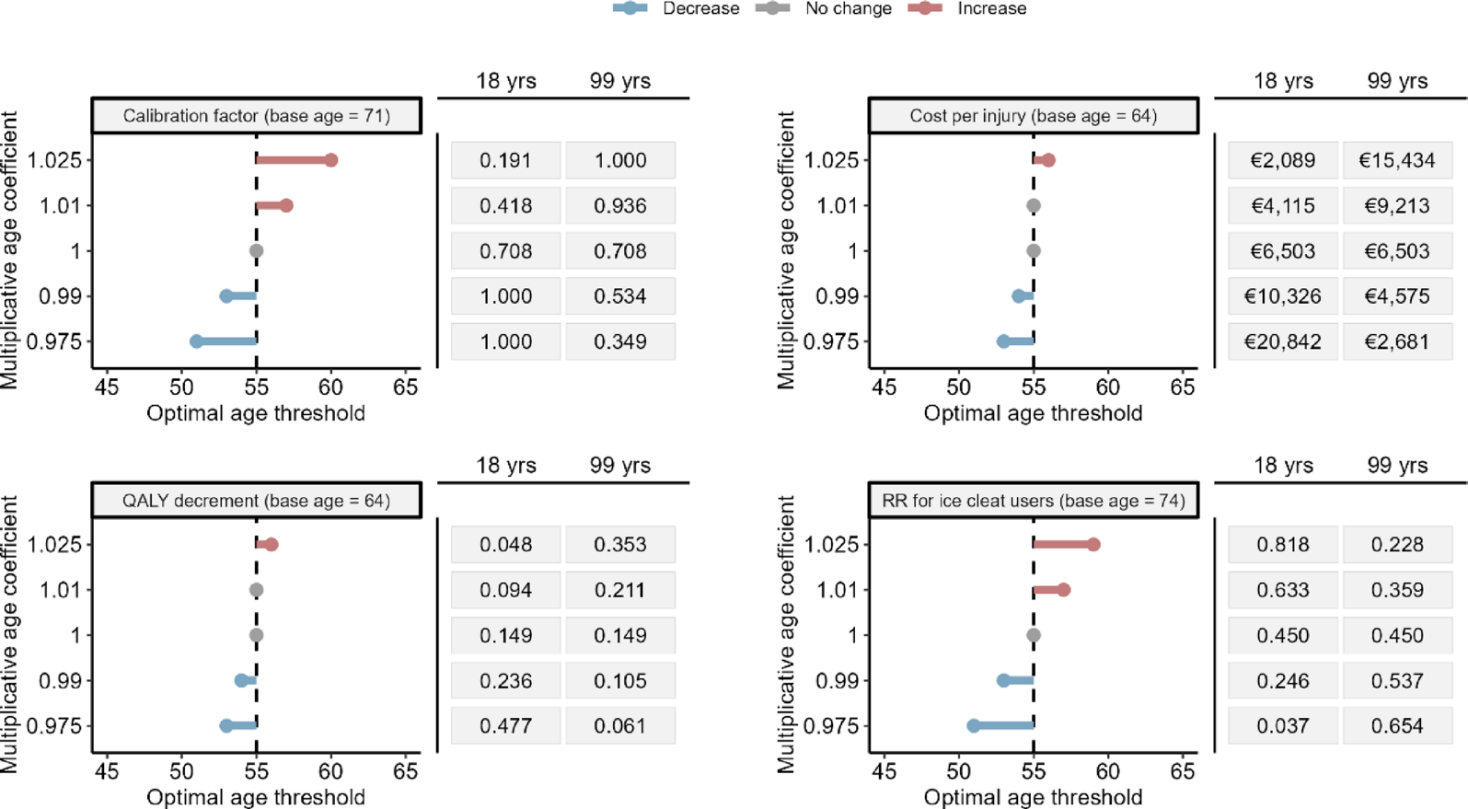
Tornado diagrams showing the optimal age thresholds from deterministic sensitivity analyses allowing for age heterogeneity in four parameters assumed constant in the base-case analysis: the compliance calibration factor, cost per injury, QALY decrement, and relative risk reduction for ice cleat use. Each analysis applies a multiplicative age effect relative to the mean age in the source data for the corresponding parameter (indicated by the base age in each panel title). The resulting parameter ranges across ages 18–99 implied by each multiplicative factor are shown to the right of each panel

Figure 4 presents tornado diagrams from the deterministic sensitivity analyses allowing for age heterogeneity in parameters assumed constant in the base-case analysis. The ranges for each parameter across ages 18–99, generated by applying the multiplicative factors (±1%, ±2.5%), are shown to the right of each panel. The±2.5% scenario represents substantial age heterogeneity, yet the estimated optimal thresholds remain largely unchanged, consistently indicating values below 65 years.

## Discussion

Public health interventions are often targeted based on perceived need, practical constraints, or political considerations, rather than formal optimization. As a result, targeting decisions may not reflect cost-effectiveness criteria, potentially leading to inefficient resource use. In Sweden, municipalities that distribute ice cleats have typically targeted adults aged 65 and older [12]. While our findings support targeted over universal distribution, it seems that limiting eligibility to older adults is unlikely to be optimal from a societal cost-effectiveness perspective.

Our base-case analysis indicates that a lower age threshold—around 55 years—is likely to be a better choice. Two mechanisms can explain this finding. First, ice- and snow-related fall injury risk begins to rise at approximately age 50 (Fig. 2B). Second, behavioral data suggest that individuals in their 50 s are beginning to form positive attitudes toward ice cleat use but have not yet adopted the behavior to the same extent as older adults (Fig. 2A). This may reflect an early stage in the behavior change process—where awareness of risk exists, but adoption has not occurred. A small nudge, such as the offer of a free pair of cleats, may be sufficient to trigger uptake in this group.

Our findings extend previous research on ice cleat distribution, which has focused exclusively on older adults [12, 14–16], by evaluating cost-effectiveness across a wider range of age thresholds. Although the exact optimal cutoff is uncertain due to structural and parameter uncertainty, the conclusion that targeting only those aged 65 and older is suboptimal appears robust. In some sensitivity analyses, no distribution is preferred—contrary to earlier cost-benefit studies [14–16]—but, as discussed further in the limitations section, several factors likely bias our estimates conservatively against distribution (e.g., the exclusion of long-term injury consequences and assumption that the programs purchase one ice cleat pair per eligible citizen).

Our results have practical implications for program design and suggest that policymakers should consider extending eligibility to younger age groups. While our analysis does not address implementation directly, previous studies indicate that these programs have generally been implemented successfully [12]. Existing initiatives often rely on infrastructure tailored to older adults, such as partnerships with pensioners’ organizations or fall prevention campaigns [12]. However, some municipalities have used distribution methods that could easily be adapted for broader populations (for example, Gothenburg mailed coupons to all citizens aged 65 and older [16]). Extending such approaches or combining them with other strategies, such as social media outreach, may help effectively reach younger adults. Our sensitivity analyses suggest that if distribution is optimized to purchase ice cleats selectively while still reaching all potential compliers, universal distribution to all adults may be an acceptable strategy. That being said, determining the most effective and equitable distribution strategy across age groups warrants further empirical investigation.

### Strengths and limitations

This study has several strengths. To our knowledge, it is the first formal evaluation of the cost-effectiveness of ice cleat distribution across age-based policy thresholds, adding new evidence to the relatively under-studied area of outdoor fall prevention. Our model is calibrated to external empirical data and reproduces observed injury reductions [14]. It also incorporates age-specific behavioral data and aligns compliance estimates with observed changes after real-world implementations to estimate realistic, age-specific policy impacts.

There are also important limitations. First, we lack age-specific estimates for several key parameters, including the relative risk reduction of ice cleat use, QALY losses, injury costs, and the compliance calibration factor. In the base-case analysis, we assume a constant relative risk reduction. Although this assumption can be questioned, risk differences generally show greater heterogeneity than ratios [31]. While injury severity tends to increase with age, all injuries in our model required outpatient or inpatient care, which may mitigate bias from applying uniform costs and QALY loss estimates. To assess the impact of these simplifications, we conducted deterministic sensitivity analyses varying age-invariant parameters by ± 1% and ± 2.5% per one-year age group relative to the mean age in their data sources, where ± 2.5% represents substantial age heterogeneity. Across all scenarios, younger thresholds than 65 + remained optimal, suggesting that the main findings are robust to plausible age variation.

Second, we lack data on the long-term consequences of pedestrian fall injuries. Pedestrian falls can lead to lasting medical disability [6], which is not captured in our model. As a result, we likely underestimate the true benefits of prevention.

Third, we rely on behavioral data from 2007 to estimate potential compliance. While these data are dated, our estimates are calibrated to observed uptake from more recent interventions [13], which should account for uniform level shifts in compliance behavior since then. However, this calibration does not capture potential secular changes in the relationship between age and compliance. Reassuringly, the deterministic sensitivity analysis allowing the compliance calibration factor to vary by age showed limited impact on the overall results.

Fourth, the base-case analysis assumes cleats are distributed uniformly to all eligible individuals, regardless of current usage. In practice, targeted outreach strategies could prioritize non-users, potentially improving cost-effectiveness and shifting the optimal age threshold downward.

Finally, we note that economic evaluations are inherently context-dependent and are intended to reflect local effect sizes and resource valuations [32]. Our study draws primarily on Swedish data, where ice-related fall injuries are common, and our results are unlikely to generalize to warmer climates. However, they might apply to countries with similar climate, resource valuation, and implementation conditions (e.g., other Nordic countries). As with all model-based economic evaluations, our findings are also contingent on assumptions and parameter choices and should be interpreted as projections. These limitations can only be addressed through empirical evaluation. A logical next step would therefore be to conduct a randomized controlled trial offering free ice cleats to individuals across different age groups, with sufficient power to detect potential effect heterogeneity.

## Conclusions

Our study suggests that targeted ice cleat distribution is more cost-effective than universal provision and that extending eligibility to middle-aged adults may be preferable to programs limited to older adults. Beyond these findings, it also illustrates how cohort models combined with behavioral data can guide targeting decisions in injury prevention programs.

## Data Availability

Injury outcome and behavioral survey data are subject to license and ethical restrictions and are not publicly available. They may be obtained from the corresponding author upon reasonable request, with approval from the Swedish Ethical Review Authority and a Data Processing Agreement with Karlstad University. Posterior parameter estimates from Bayesian models requiring these data are provided, allowing the cohort model to be fully reproduced using these estimates together with public data and code available at the Open Science Framework (https://osf.io/2qdgh/).

## Appendix

We here provide a technical description of the cohort model. We develop a model that accounts for age-specific injury risks and ice cleat behaviors, updating population size, cleat use, fall‑injury risk, costs, and quality-adjusted life years in one‑year cycles. The simulation starts with a closed cohort of $\:N$ individuals aged 18–99 + according to the national age distribution, normalized to sum to one, which gives a per-capita interpretation of all cost-effectiveness outputs. Our cohort model starts at age 18, denoted by $\:{a}_{0}$, and we run the model until 99% of that sub‑cohort has died. We consider a simple distribution program where distribution occurs only once (at the initial cycle) for everyone aged​ $\:\ge\:d$ (the program’s eligibility threshold).

### Cohort survival model

Let

$$\:\mathbf{N}\left(k\right)={\left({N}_{0}\left(k\right),\dots\:,{N}_{J-1}\left(k\right)\right)}^{T}$$

be the numbers alive in cycle *k* across *J* one-year age states ($\:i=0,\dots\:,J-2$ for ages $\:{a}_{0}+i$ and $\:i=J-1$ for 99+). With age-specific annual mortality risks $\:{q}_{i}$ we define survival probabilities $\:{s}_{i}=1-{q}_{i}$. One year of ageing and mortality is represented by the sparse transition matrix $\:T$ whose only non-zero elements are

$$\:{T}_{i+1,i}={s}_{i},\:\:{T}_{J-1,J-1}={s}_{J-1}.$$

This means that survivors move down one age bracket each cycle, except in the 99 + state where they remain recorded as 99+, and that the cohort evolves according to the standard Markov relation

$$\:\mathbf{N}\left(k\right)={T}^{k}\mathbf{N}\left(0\right),$$

where $\:{T}^{k}$ denotes the *k*-th power of $\:T$ and $\:\mathbf{N}\left(0\right)$ is the vector of initial population sizes.

### Behavioral model

Following the local average treatment‑effect framework stratify the initial cohort to one of three latent types: always‑users (use cleats regardless), never‑users, and compliers (use cleats only if offered). Only compliers change behavior when distribution is introduced at the initial cycle ($\:k=0$). Let $\:{\mathbf{N}}^{A}\left(k\right)$, $\:{\mathbf{N}}^{N}\left(k\right)$ and $\:{\mathbf{N}}^{C}\left(k\right)$ represent the number of always-takers, never-takers, and compliers at cycle *k*, satisfying $\:\mathbf{N}\left(k\right)={\mathbf{N}}^{A}\left(k\right)+{\mathbf{N}}^{N}\left(k\right)+{\mathbf{N}}^{C}\left(k\right)$.

We obtain the natural number of ice cleat users (without distribution) by taking the element-wise (Hadamard) product of $\:\mathbf{N}\left(k\right)$ and the vector of age-specific proportions of always-takers in the cohort, $\:{\mathbf{p}}^{A}={\left({p}_{0}^{A},\dots\:,\:{p}_{J-1}^{A}\right)}^{T}$,

$$\:{\mathbf{N}}^{A}\left(k\right)=\mathbf{N}\left(k\right)\circ\:{\mathbf{p}}^{A}.$$

In our simulation, distribution occurs only once (at cycle $\:k=0$). The complier population is realized at this point only and only for ages $\:\ge\:d$, given their age-specific compliance probabilities at baseline. The number of *realized* compliers at cycle *k* is therefore given by.

$$\:{\mathbf{N}}_{r}^{C}\left(k\right)={T}^{k}{\mathbf{N}}^{C}\left(0\right)$$

$$\:={T}^{k}\left(\mathbf{N}\left(0\right)\circ\:{\mathbf{p}}^{C}\circ\:\mathbf{d}\right),$$

where $\:{\mathbf{p}}^{C}$ is the vector of age-specific proportions of compliers in the cohort and $\:\mathbf{d}$ is a corresponding vector coded as 1 for ages $\:\ge\:d$ and zero otherwise. The total number of users at cycle *k* in our ice cleat distribution arm is therefore given by.

$$\:{\mathbf{N}}^{U}\left(k\right)={\mathbf{N}}^{A}\left(k\right)+{\mathbf{N}}_{r}^{C}\left(k\right)$$

$$\:=\mathbf{N}\left(k\right)\circ\:{\mathbf{p}}^{A}+{T}^{k}\left(\mathbf{N}\left(0\right)\circ\:{\mathbf{p}}^{C}\circ\:\mathbf{d}\right).$$

To model subsiding compliance over time, we only count individuals still alive in the compliance sub-cohort up to a given number of cycles as cleat users (see main text for details).

### Outcome model

Let $\:{\mathbf{r}}^{1}={\left({r}_{0}^{1},\dots\:,{r}_{J-1}^{1}\right)}^{T}$ be a vector of age-specific, potential annual injury risks due to ice-related falls if, perhaps contrary to fact, the entire cohort uses ice cleats, and $\:{\mathbf{r}}^{0}$ be a similar vector for the potential risks if none of them do. Under standard counterfactual consistency assumptions, including no spillover effects, these quantities are related to the realized risk vector $\:{\mathbf{r}}^{obs}$ as follows:

$$\:{\mathbf{r}}^{obs}=\left({\mathbf{p}}^{A}\circ\:{\mathbf{r}}^{1}\right)+\left(\left(1-{\mathbf{p}}^{A}\right)\circ\:{\mathbf{r}}^{0}\right),$$

where $\:{\mathbf{p}}^{A}$ is the vector of age-specific always-user shares. We observe $\:{\mathbf{r}}^{obs}$, $\:{\mathbf{p}}^{\varvec{U}}$, and have access to causal relative risk estimates for ice cleat use,$\:\:RR$, assumed here for lack of age-specific data to be constant across all ages.

Because $\:{\text{r}}_{i}^{1}={RR\text{r}}_{i}^{0},$ we have that.

$$\:{\text{r}}_{\varvec{i}}^{obs}={\text{p}}_{\varvec{i}}^{A}\left({RR\text{r}}_{i}^{0}\right)+\left(\left(1-{\text{p}}_{i}^{A}\right){\text{r}}_{i}^{0}\right)$$

1 $$\:=\left(RR{p}_{i}^{A}+1-{p}_{i}^{A}\right){r}_{i}^{0}.$$

We exploit this equivalence to estimate the potential risks for non-ice-cleat-users:

$$\:{r}_{i}^{0}=\frac{{r}_{i}^{obs}}{RR{p}_{i}^{A}+1-{p}_{i}^{A}}\Rightarrow\:{\mathbf{r}}^{0}={\mathbf{r}}^{obs}\oslash\:\left(RR{\mathbf{p}}^{A}+(1-{\mathbf{p}}^{A})\right),$$

where $\:\oslash\:$ represents element-wise division, and compute the potential risks for ice cleat users as

$$\:{\mathbf{r}}^{1}={RR\mathbf{r}}^{0}.$$

The total expected number of ice-related injuries in cycle *k* is then given by

$$\:Y\left(k\right)={\mathbf{r}}^{1}\bullet\:{\mathbf{N}}^{U}\left(k\right)+{\mathbf{r}}^{0}\bullet\:\left(\mathbf{N}\left(k\right)-{\mathbf{N}}^{U}\left(k\right)\right),$$

where $\:\bullet\:$ is the dot product. Costs and QALY losses per injury (see Table 1) are then applied to $\:Y\left(k\right)$; totals are the sum over cycles, discounted at 3% per annum in the base-case.

## Abbreviations

HBM: Health Belief Model
ICD-10: International Statistical Classification of Diseases and Related Health Problems, 10th revision
INLA: Integrated Nested Laplace Approximation
NMB: Net monetary benefit
QALY: Quality-adjusted life year
SEK: Swedish krona
